# Plasma metabolomics supports non-fasted sampling for metabolic profiling across a spectrum of glucose tolerance in the Nile rat model for type 2 diabetes

**DOI:** 10.1038/s41684-023-01268-0

**Published:** 2023-10-19

**Authors:** Benton J. Anderson, Anne M. Curtis, Annie Jen, James A. Thomson, Dennis O. Clegg, Peng Jiang, Joshua J. Coon, Katherine A. Overmyer, Huishi Toh

**Affiliations:** 1 Department of Chemistry, University of Wisconsin-Madison, Madison, WI USA; 2 Department of Molecular, Cellular, and Developmental Biology, University of California, Santa Barbara, CA USA; 3 Neuroscience Research Institute, University of California, Santa Barbara, CA USA; 4 Department of Biomolecular Chemistry, University of Wisconsin-Madison, Madison, WI USA; 5 Morgridge Institute for Research, Madison, WI USA; 6 Department of Biological, Geological and Environmental Sciences, Cleveland State University, Cleveland, OH USA; 7 Center for Gene Regulation in Health and Disease, Cleveland State University, Cleveland, OH USA; 8 Center for RNA Science and Therapeutics, School of Medicine, Case Western Reserve University, Cleveland, OH USA

**Keywords:** Machine learning, Metabolomics, Diabetes, Predictive markers, Mass spectrometry

## Abstract

Type 2 diabetes is a challenge in modern healthcare, and animal models are necessary to identify underlying mechanisms. The Nile rat (*Arvicanthis niloticus*) develops diet-induced diabetes rapidly on a conventional rodent chow diet without genetic or chemical manipulation. Unlike common laboratory models, the outbred Nile rat model is diurnal and has a wide range of overt diabetes onset and diabetes progression patterns in both sexes, better mimicking the heterogeneous diabetic phenotype in humans. While fasted blood glucose has historically been used to monitor diabetic progression, postprandial blood glucose is more sensitive to the initial stages of diabetes. However, there is a long-held assumption that ad libitum feeding in rodent models leads to increased variance, thus masking diabetes-related metabolic changes in the plasma. Here we compared repeatability within triplicates of non-fasted or fasted plasma samples and assessed metabolic changes relevant to glucose tolerance in fasted and non-fasted plasma of 8–10-week-old male Nile rats. We used liquid chromatography–mass spectrometry lipidomics and polar metabolomics to measure relative metabolite abundances in the plasma samples. We found that, compared to fasted metabolites, non-fasted plasma metabolites are not only more strongly associated with glucose tolerance on the basis of unsupervised clustering and elastic net regression model, but also have a lower replicate variance. Between the two sampling groups, we detected 66 non-fasted metabolites and 32 fasted metabolites that were associated with glucose tolerance using a combined approach with multivariable elastic net and individual metabolite linear models. Further, to test if metabolite replicate variance is affected by age and sex, we measured non-fasted replicate variance in a cohort of mature 30-week-old male and female Nile rats. Our results support using non-fasted plasma metabolomics to study glucose tolerance in Nile rats across the progression of diabetes.

## Abstract (web summary)

The authors used liquid chromatography–mass spectrometry to compare the metabolic profile of non-fasted and fasted plasma samples in the Nile rat model of type 2 diabetes. Metabolite measurements in non-fasted samples were more reproducible than in fasted samples, supporting the use of non-fasted plasma metabolomics to study glucose tolerance in Nile rats during diabetes progression.

## Main

Diabetes is an urgent global health challenge with an accelerating incidence rate in recent decades. Currently, 537 million adults are living with diabetes and 541 million adults have impaired glucose tolerance with a high risk of developing type 2 diabetes^1^. To better understand metabolic changes associated with impaired glucose tolerance, we need suitable animal models and experimental methods that can capture these changes.

The Nile rat (*Arvicanthis niloticus*) is a model of type 2 diabetes with key benefits over other rodent models. First, diabetes is rapidly induced in both sexes by conventional laboratory rodent chow that is hypercaloric for the Nile rat compared to its native fiber-rich diet^2,3^. On conventional rodent chow, the onset of diabetes can range from a month to a year of age, and by 6 months of age, most of the Nile rats would have developed diabetes^4^. By contrast, common laboratory mice and rats are relatively resistant to diet-alone induced diabetes, and additional chemical or genetic manipulations are used to promote diabetes^5^. Second, diabetic Nile rats can develop long-term diabetic complications mimicking clinical features of patients with diabetes^6–8^, including diabetic retinopathy^9,10^. Third, the Nile rat model is outbred and displays a wide range of diabetic phenotypes^11^, reflecting its underlying genetic diversity. Fourth, the Nile rats, like humans, are active during the day^12^, unlike common nocturnal rodent models. Additionally, the Nile rat has a reference genome for mechanistic studies^13^. Overall, the Nile rat is highly suited to study the underlying mechanisms of glucose intolerance in diet-induced diabetes.

When considering experimental methods for studying diabetes, a majority of studies looking for metabolic changes will use blood that has been sampled under fasted state to avoid excess variability from unrestricted eating behavior. However, for the early progression of diabetes, it is known that postprandial hyperglycemia precedes fasted hyperglycemia, and thus is a more sensitive measurement for early diabetes, as demonstrated in human studies^14–16^ and in the Nile rat model^11,17–19^. In addition, there is some evidence that the postprandial state might be associated with reduced variability in blood metabolites^20^. For rodent models, non-fasted state probably represents a postprandial state given the high frequency of food intake. Yet, so far, no study has validated the use of non-fasted sampling for metabolomics studies in rodent models. Specifically, reproducibility and replicate variability between fasted and non-fasted states have not been sufficiently analyzed. Therefore, this study compares metabolite variance between non-fasted and fasted blood sampling for studying progressive glucose intolerance in Nile rats.

To investigate the metabolic differences between the fasted state and non-fasted state, we performed metabolomics using liquid chromatography coupled to mass spectrometry (LC–MS) to measure a broad range of plasma biomolecules^21^. LC–MS has been used previously to analyze variance of plasma sampling across metabolites^20,22–25^ and to detect plasma biomarkers relevant to diabetes in mice and humans^26,27^. To assess replicate variance in fasted versus non-fasted samples and to capture markers of diabetes, we measured metabolites in non-fasted and fasted plasma samples in triplicate. The cohort consisted of male Nile rats aged 8–10 weeks displaying varied levels of glucose tolerance—from non-diabetic to overtly diabetic—at 12 weeks old. We found that metabolites in non-fasted plasma sampling had better predictive power of impaired glucose tolerance. Counter to the accepted wisdom that ad libitum feeding leads to increased variability, we found that metabolites in non-fasted samples had lower median replicate variance than metabolites in fasted samples. To validate these results, we used a different age group of Nile rats of both sexes and assessed metabolite variance in triplicate non-fasted plasma samples. In this validation cohort, replicate variance of metabolites was similar or lower than that found in the primary cohort. Our data supports the use of a non-fasted state for plasma sampling in metabolic studies of Nile rats due to better association to glucose tolerance levels and lower replicate variance independent of age or sex.

In this Article, we performed plasma metabolomics to compare the non-fasted versus fasted state in Nile rats. We employed a study design in which plasma was sampled in both states for each Nile rat, and we investigated how metabolite abundances and variance were affected by glucose tolerance and plasma sampling method. We concluded that plasma metabolomics using non-fasted sampling is valuable for studying diabetes across a spectrum of glucose tolerance in Nile rats.

## Results

### Metabolomic profiling of non-fasted and fasted plasma associated with glucose tolerance

Nile rats have been well described to develop glucose intolerance when consuming conventional rodent chow 5008, in part due to the glycemic load of 5008 (refs. ^11,17–19,28^). Additionally, males progress toward glucose intolerance more rapidly than females on the same dietary challenge^28^. Because the Nile rat model is genetically diverse, they display a spectrum of glucose intolerance. To identify plasma metabolites that show trends over a spectrum of glucose tolerance, we analyzed the plasma of juvenile males fed rodent chow.

**Fig. 1:**
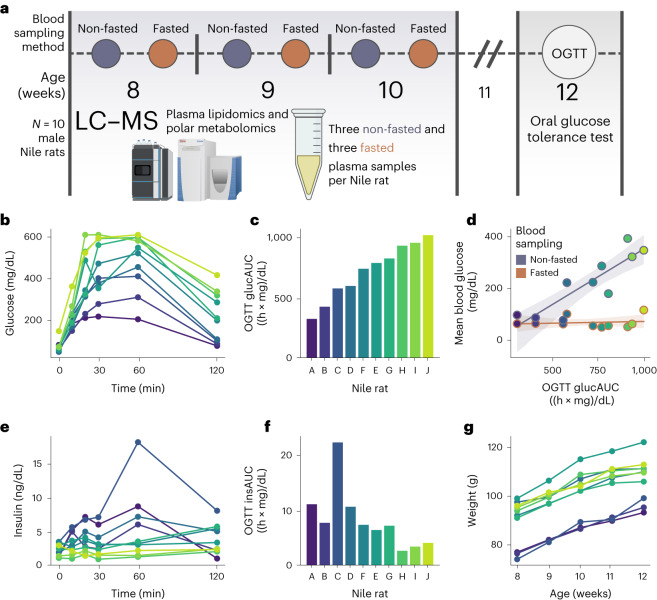
Metabolomic study design and characterization of glucose tolerance. **a**, Overview of study design with analysis of plasma metabolites and lipids in ten male Nile rats at 8–10 weeks in fasted and non-fasted conditions, and measurement of glucose tolerance by OGTT at 12 weeks. **b**, OGTT curve with blood glucose levels taken at 0, 10, 20, 30, 60 and 120 min after ingesting glucose delivered by gavage. **c**, Area under the curve calculated from glucose OGTT (OGTT glucAUC). Nile rats are ordered by increasing OGTT glucAUC. **d**, The correlation between OGTT glucAUC at 12 weeks and mean blood glucose levels at 8–10 weeks, measured in fasted versus non-fasted state of the Nile rats. Shaded region depicts the 95% bootstrapped confidence interval. **e**, OGTT curve with plasma insulin levels taken at 0, 10, 20, 30, 60 and 120 min after ingesting glucose. **f**, Area under the curve calculated from insulin OGTT (OGTT insAUC). Nile rats are ordered by increasing OGTT glucAUC. **g**, Growth chart from 8 to 12 weeks based on whole body weight for Nile rats A to J.

Specifically, to evaluate replicate variance between non-fasted and fasted plasma samples, we collected fasted and non-fasted samples in ten male Nile rats, which were taken at 8, 9 and 10 weeks of age (60 samples in total; Supplementary Data Tables 1 and 2); and later, an oral glucose tolerance test (OGTT) was performed at 12 weeks (Fig. 1a). Time of day of sampling for the non-fasted state was found to have no significant effect on the observed blood glucose value (Supplementary Fig. 1a,b). Sampling the plasma at least 2 weeks before OGTT enabled the animals to recover from weekly blood collections before OGTT. Figure 1b shows the glucose excursion across 2 h during the OGTT. Nile rats labeled A to J are ordered on the basis of area under the curve of glucose levels during OGTT (OGTT glucAUC) (Fig. 1c) and show a range of glucose tolerance evenly distributed across these ten Nile rats. Within the range of glucose tolerances captured in our study cohort, random blood glucose (RBG) exhibited a positive trend with subsequent OGTT glucAUC whereas there was no association to fasted blood glucose (FBG) (Fig. 1d).

Additionally, we measured blood insulin concentration during the OGTT. In humans, patterns of insulin concentration during OGTT can predict incident type 2 diabetes^29^. Here we observed similar patterns where the healthier Nile rats A to D had higher insulin levels at 60 min than 120 min, compared to Nile rats E to G (Fig. 1e). Notably, the area under the curve from plasma insulin during OGTT (OGTT insAUC) was exceptionally high in Nile rat C (Fig. 1f). This hyperinsulinemic response suggests that Nile rat C was at a pre-diabetic or at an early stage of diabetes. Conversely, Nile rats H, I and J were hypoinsulinemic, indicating that these rats were more advanced in the diabetes spectrum. Weekly body weight (Fig. 1g) and blood glucose (Supplementary Fig. 1c) were measured from weeks 8 to 12. Though the growth rates were similar, the initial weights taken at 8 weeks segregated the animals into two groups, with A, B and C at lower weights and D through J at higher weights. Based on 8-week RBG and FBG, seven Nile rats had non-fasted hyperglycemia (RBG >200 mg/dL in Nile rats D to J) and two Nile rats had fasted hyperglycemia (FBG >126 mg/dL in Nile rats D and J).

### Unsupervised clustering reveals better association of metabolite abundance to glucose tolerance in non-fasted plasma

**Fig. 2:**
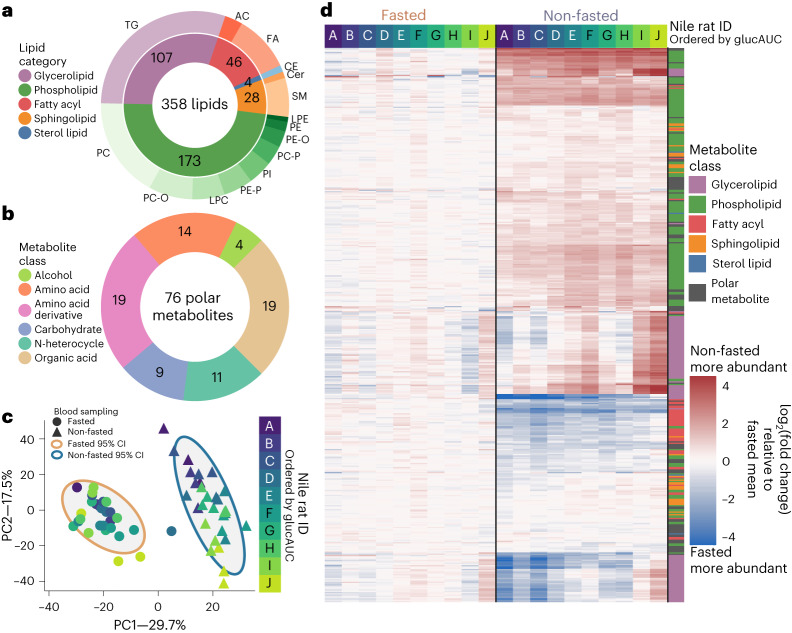
Plasma lipids and polar metabolites separate non-fasted and fasted sampling methods in unsupervised clustering. **a**, A summary of counts of 358 identified lipids, divided into 5 lipid categories on the inner circle, and lipid class on the outer circle. **b**, A summary of counts of 76 total identified polar metabolites from 6 classes. **c**, PCA of all non-fasted and fasted plasma samples using all features from both HILIC and reversed phase LC–MS methods. Principal components 95% confidence intervals (CI) are displayed around non-fasted and fasted points. Points are colored according to Nile rat ID, with color gradation based on OGTT glucose AUC order. **d**, Heat map of all identified lipids and polar metabolites, separated by fasted (left) and non-fasted (right) conditions. Each column is the mean of triplicate Nile rat samples collected at 8, 9 and 10 weeks. Rows are ordered on the basis of clustering (Methods) using non-fasted plasma samples. Lipid or polar metabolite indicator are given in colorbar. Metabolite abundances are given as log_2_ fold change of the difference from mean of fasted samples. Cer, ceramide; FA, fatty acyl; LPC, lysoPC; LPE, lysoPE; PC, phosphatidylcholine (-P plasmenyl; -O plasmanyl); PI, phosphatidylinositol.

To characterize the plasma biomolecules in these Nile rats under fasted and non-fasted conditions, we performed discovery metabolomics and lipidomics by LC–MS/MS, and calculated relative quantification by integrating chromatographic peak area. We annotated 358 lipids across 5 lipid categories^26,30^, including glycerolipids, phospholipids, sphingolipids, fatty acyls and sterol lipids; 556 lipid chromatographic features remained unannotated but were included in some of the downstream analyses (Fig. 2a and Supplementary Data Tables 3 and 7). Of the annotated lipids, 200 were identified at species level and 158 were identified at molecular species level^31^. Among polar metabolites, we annotated 76 compounds from 6 compound classes, including organic alcohols, amino acids (AAs), AA derivatives, nitrogen heterocycles, carbohydrates and organic acids (Fig. 2b). A total of 419 polar metabolite features remained unannotated.

Next we performed principal component analysis (PCA) using all metabolite features in our 60 plasma samples. PCA revealed two clusters separated on the first principal component by fasted or non-fasted sampling conditions (Fig. 2c). Within the non-fasted cluster, the samples seem to be ordered by OGTT glucAUC along the second principal component, whereas a similar ordering is absent in the fasted cluster. This suggests that non-fasted metabolomic changes are associated with glucose tolerance.

To further explore high-level trends in plasma metabolites, we constructed a heat map ordered by Nile rat OGTT glucAUC on the columns, with hierarchical clustering of annotated metabolites on the rows (Fig. 2d). From here on, we refer to plasma samples collected in the non-fasted or fasted state as ‘non-fasted samples’ or ‘fasted samples’, respectively. Overall, non-fasted samples display greater log_2_ fold changes relative to mean metabolite abundance in fasted samples. In general, the lipids seem to have a larger dynamic range than the polar metabolites. Glycerolipids show the most apparent trends in association to OGTT glucAUC ranking.

### Non-fasted Nile rat plasma yields lower replicate variance across metabolites

**Fig. 3:**
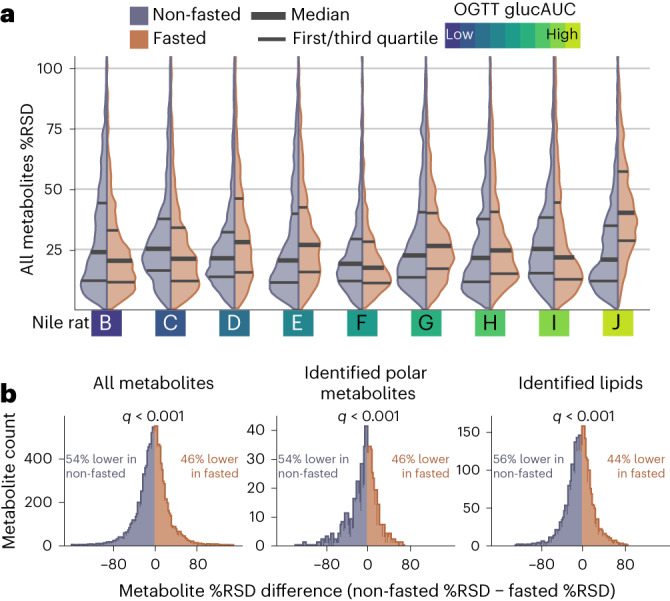
Non-fasted plasma metabolites show similar variance within Nile rats and lower variance within each metabolite compared to fasted sampling. **a**, Comparison of %RSD distributions for non-fasted and fasted plasma metabolites within each Nile rat across triplicate plasma sampling. A total of five out of nine Nile rats show lower median %RSD in non-fasted sampling. Overall median %RSD in non-fasted sampling is 22.2%, compared to overall median %RSD of 24.9% in fasted sampling. Nile rats are ordered on the basis of OGTT glucAUC, with more glucose-intolerant Niles rats to the right. **b**, Calculating %RSD in each sampling method and then subtracting non-fasted %RSD from fasted %RSD for each metabolite in all Nile rats yields percentage point differences. Distribution of percentage point differences are shown, where the blue portion on the left are metabolites whose %RSD difference is less than 0%, that is, it has a lower %RSD in non-fasted metabolites. Across all metabolites, 54% of matched measurements have lower non-fasted %RSD. Across identified polar metabolites, 54% are lower in non-fasted samples and in identified lipids, and 56% are lower in non-fasted samples. Significance testing using Wilcoxon signed rank test reveals that the median for each distribution significantly differs from 0% (*q* < 0.001 for all three distributions).

A major concern of using non-fasted plasma samples is the excess variability driven by ad libitum feeding and varying degrees of postprandial state. To assess plasma metabolite variability within replicate samples between fasted and non-fasted sampling states, we calculated the percent relative standard deviation^32^ (%RSD) across an individual’s triplicate of 8–10-week plasma samples (Supplementary Data Table 5). The distribution %RSDs for all metabolites is shown for each Nile rat in Fig. 3a, grouped by sampling method. We excluded Nile rat A, which had only two out of three replicate fasted samples. Of the remaining nine animals, five had lower median metabolite %RSD in non-fasted replicates. The median %RSD across all triplicate metabolite measurements was smaller in non-fasted samples (22.2%) compared to fasted samples (24.9%). At an individual metabolite level, we calculated the percentage point difference between non-fasted and fasted %RSD for each metabolite per Nile rat and show the distribution of these paired differences in Fig. 3b. A larger number of %RSD differences were lower in non-fasted replicates for all metabolites (54%), identified polar metabolites (54%) and identified lipids (56%). All three groups show significant difference from 0 percentage point difference (*q* < 0.001; Methods). Similar analysis for other groups based on metabolite class, lipid class and lipid category are given in Supplementary Fig. 2. A total of 8 out of 15 lipid classes (LysoPC, phosphatidylcholine (PC), sphingomyelin (SM), plasmenyl-PC, plasmanyl-PC, plasmenyl-phosphatidylethanolamine (PE), plasmanyl-PE and triacylglycerol (TG)) had significantly lower (*q* < 0.05) %RSDs in non-fasted replicates. Among polar metabolite groups, carbohydrates, organic acids and AAs yielded significantly lower %RSDs in non-fasted replicates. Additionally, across all groupings metabolites tested, none showed a significantly lower %RSD in fasted replicates, indicating that non-fasted samples have lower replicate variance. These results are supported by a similar analysis using proton nuclear magnetic resonance metabolomics on postprandial versus fasted human plasma samples^20^.

### Non-fasted samples are superior to fasted plasma samples for predicting OGTT glucAUC in young males

**Fig. 4:**
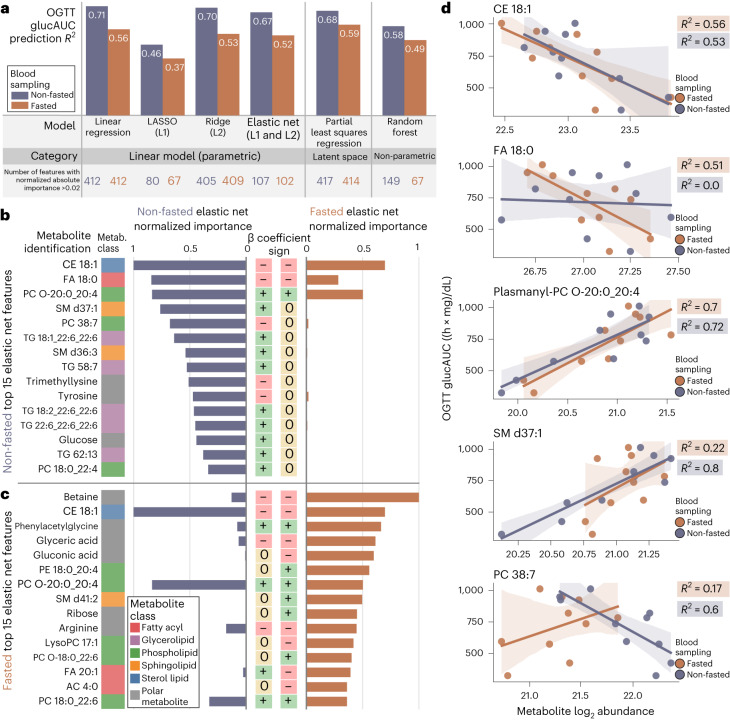
Linear regression modeling trained on non-fasted plasma samples achieves superior performance in predicting OGTT glucose AUC. **a**, Median *R*^2^ was compared for six different machine learning model architectures trained on non-fasted and fasted plasma metabolite abundances. Across all models, non-fasted data provided higher median *R*^2^ values. Linear regression returned the highest *R*^2^, but regularized linear models (LASSO, ridge and elastic net) and other models (PLSr and random forest) were trained to perform feature shrinkage or reduction of feature space dimensionality. Elastic net was most performant for biological interpretation due to its nearly equivalent *R*^2^ to linear regression and large coefficient shrinkage. The six models were categorized on the basis of the underlying mechanism of prediction, divided between parametric, latent space and non-parametric methods. **b**,**c**, The importance values of the top 15 metabolites in non-fasted (**b**) and top 15 metabolites in fasted (**c**) elastic net modeling presented along with importance of molecule in the other sampling method. CE 18:1 and PC O-20:0_20:4 are bolded due to presence in both top 15 lists. **d**, The top five most important metabolites from non-fasted elastic net modeling were individually regressed to OGTT glucAUC. Dots represent the mean value of each Nile rat’s triplicate metabolite abundance; shaded regions are the 95% bootstrapped confidence interval.

Earlier, we suggested that non-fasted plasma samples show stronger associations to OGTT glucAUC compared to fasted samples based on unsupervised modeling with PCA (Fig. 2c). To test this hypothesis, we trained regression models to learn potential metabolite associations to glucose tolerance (Fig. 4a and Supplementary Data Table 9). Linear regression, least absolute shrinkage and selection operator (LASSO), ridge, elastic net, partial least squares regression (PLSr) and random forest machine learning models were trained to predict 12-week OGTT glucAUC using all annotated lipids and polar plasma metabolites sampled at age 8–10 weeks. We trained competing models using non-fasted versus fasted plasma samples. Model performance was assessed using the median coefficient of determination (*R*^2^). Overall, the models trained on non-fasted data yielded a higher median *R*^2^ over the same model trained on fasted data. While linear regression was the most performant (*R*^2^ = 0.71 non-fasted, *R*^2^ = 0.56 fasted), biological interpretation of its learned parameters is complicated by the large number of metabolite features retained in the model. The number of features can be minimized by methods such as regularization in linear modeling using LASSO, ridge or elastic net^33^, bootstrapping in random forests^34^, or transformation into lower-dimensional latent spaces in PLSr^35^. Of these five model types, elastic net achieved both high performance (*R*^2^ = 0.67 non-fasted, *R*^2^ = 0.52 fasted) and substantial coefficient shrinkage (107 and 102 features with normalized absolute importance >0.02 in non-fasted and fasted, respectively). Compared to other methods, LASSO (*R*^2^ = 0.46 non-fasted, *R*^2^ = 0.37 fasted) and random forest (*R*^2^ = 0.58 non-fasted, *R*^2^ = 0.49 fasted) had lower performance. Ridge and PLSr achieved slightly higher *R*^2^ than elastic net, but failed to shrink the number of important metabolite features compared to elastic net. Therefore, elastic net was selected as the optimal model.

The top 15 most important metabolites for predicting OGTT glucAUC in non-fasted and fasted elastic net models are shown in Fig. 4b,c. There is sparse overlap between the top 15 metabolites in the non-fasted and fasted samples, except for cholesteryl ester (CE) 18:1 and plasmanyl-PC O-20:0_20:4. Critically, a high importance in a multivariate model such as elastic net does not ensure that the metabolite predicts OGTT glucAUC well in a univariate model. To demonstrate the performance of univariate prediction, we show the linear regression results of predicting OGTT glucAUC from the top five elastic net non-fasted features (Fig. 4d). CE 18:1 and plasmanyl-PC O-20:0_20:4 are both in the top 15 fasted and non-fasted elastic net metabolites and achieve approximately similar *R*^2^ in both fasted and fed models. By contrast, despite being the second ranked metabolite in non-fasted samples, FA 18:0 achieves an *R*^2^ of 0.0 in predicting OGTT glucAUC. SM d37:1 achieves *R*^2^ of 0.8 and 0.22 in non-fasted and fasted samples respectively. Superior predictive performance by the SM d37:1 model compared to the full elastic net model is due to no cross-validation. Finally, PC 38:7 returns better *R*^2^ in non-fasted samples (0.6) and displays a positive correlation, whereas in fasted samples, it shows negative to no correlation. In summary, some metabolites are useful in a multivariate model by combining their information with other metabolites to boost OGTT glucAUC prediction performance.

### Non-fasted plasma samples have more metabolites with strong associations to glucose tolerance

**Fig. 5:**
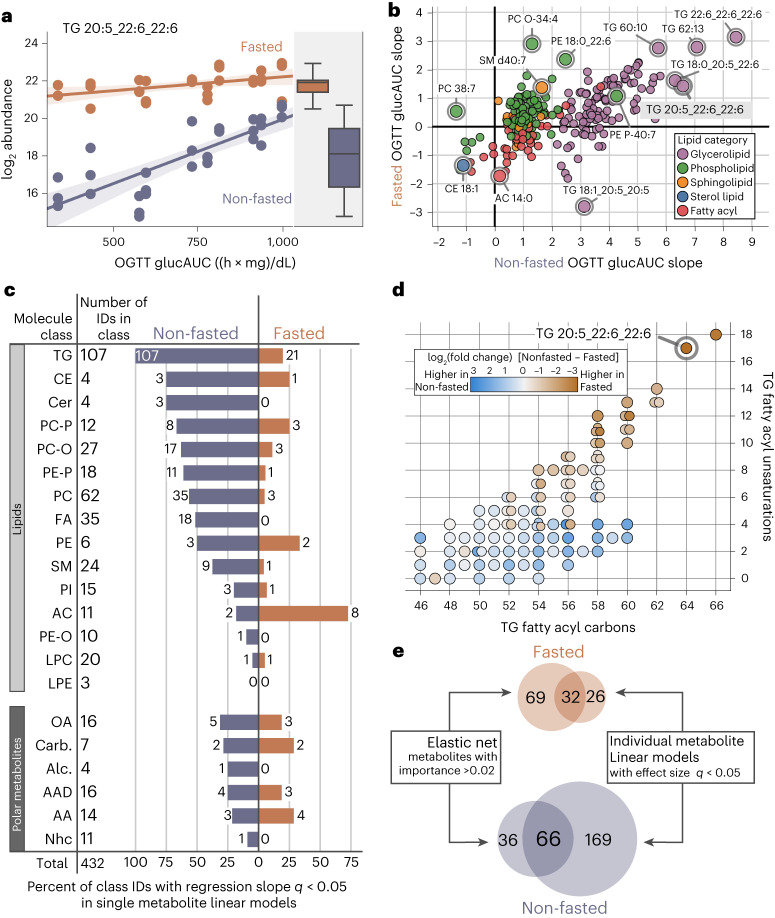
Individual metabolite linear modeling is a method for determining plasma metabolite importance in predicting glucose tolerances in Nile rats. **a**, Linear regression modeling of log2 abundance versus OGTT glucAUC for TG 20:5_22:6_22:6 within each blood sampling method shows higher mean abundance in fasted samples and a steeper regression slope in non-fasted samples. **b**, Plot of regression effect size of OGTT glucAUC versus log_2_ abundance for all identified lipids in fasted versus non-fasted samples. Lipids with the steepest slope are dominated by TGs containing PUFAs in non-fasted sampling. **c**, Linear regression modeling of log_2_ abundance versus OGTT glucAUC provides regression effect sizes and associated *q* values. The number of metabolites in each molecule class with *q* < 0.05 are shown for both non-fasted and fasted sampling. Across all metabolite classes except AC and AA, non-fasted samples have more metabolites with significant OGTT glucAUC regression slope than fasted samples. **d**, TGs are separated based on fatty acyl carbon count and fatty acyl unsaturation count, with points colored by the mean difference in log2 abundance between non-fasted and fasted. TGs with both high fatty acyl carbons (>57) and high unsaturations (>9) are more abundant in fasted plasma samples. TGs with lower unsaturation counts tend to be more abundant in non-fasted samples. TGs with identical fatty acyl carbon and unsaturation counts are shown as overlapping dots. **e**, Filtering metabolites for importance in association with OGTT glucAUC in non-fasted and fasted elastic net models (from Fig. 4) and individual metabolite models yields 66 metabolites in non-fasted sampling and 32 metabolites in fasted sampling. AAD, amino acid derivative; Alc, alcohol; Carb, carbohydrate; Nhc, nitrogen-heterocycle; OA, organic acid.

We have therefore discovered metabolites that best predicted glucose tolerance in a multivariate model setting (Fig. 4). Next, we determined which metabolites had individual associations to OGTT glucAUC. Using linear models at the individual metabolite level (Methods), we calculated the effect size of OGTT glucAUC and the interaction between sampling conditions and OGTT glucAUC. An example of the analysis is highlighted in Fig. 5a, where the abundance of TG 20:5_22:6_22:6 significantly increases with OGTT glucAUC in non-fasted samples (*q* < 0.0001), but is not significant in fasted samples (*q* = 0.058). The OGTT glucAUC effect size is greater in non-fasted than in fasted samples (*q* = 1.8 × 10^−22^). The mean abundance of TG 20:5_22:6_22:6 is significantly greater in fasted samples compared to non-fasted samples (*q* < 0.0001) (Supplementary Fig. 3a,b). Results for all metabolites are given in Supplementary Data Table 5.

To explore the OGTT glucAUC effect size of non-fasted and fasted sampling for all metabolites, we plotted regression slopes for all annotated lipids (Fig. 5b) and polar metabolites (Supplementary Fig. 3c). The abundance of all TGs was positively associated with OGTT glucAUC in non-fasted sampling, while TGs in fasted sampling had both positive and negative associations to OGTT glucAUC. For all TGs, non-fasted sampling had a steeper regression slope than fasted sampling, indicating stronger associations to glucose tolerance. The TGs most positively associated with OGTT glucAUC were TG 22:6_22:6_22:6 (66:18), TG 62:13, TG 18:0_20:5_22:6 (60:11), TG 60:10 and TG 20:5_22:6_22:6 (64:17), all of which contain polyunsaturated fatty acyls (PUFAs) such as docosahexaenoic acid (22:6*n*-3). Among lipids that were positively associated with OGTT glucAUC in both sampling methods were PC O-34:4, PE 18:0_22:6, PE 18:0_20:4, SM d40:7 and PE P-40:7. By contrast, CE 18:1 was negatively associated with OGTT glucAUC in both fasted and non-fasted sampling conditions. Overall, there were more significant metabolite associations to OGTT glucAUC in non-fasted sampling compared to fasted sampling (Fig. 5c). Across metabolite classes, only acylcarnitines (ACs) and AAs had a greater number of metabolites that were significantly associated to OGTT glucAUC in fasted sampling.

Given the strong associations between numerous TGs and OGTT glucAUC, we explored TGs further by plotting TGs separated by fatty acyl carbon count and number of unsaturations, with dots colored by log_2_ fold change between non-fasted and fasted sampling (Fig. 5d). TGs with higher carbon counts and number of unsaturations tended to be more abundant in fasted samples, whereas saturated, monounsaturated and TGs with three to four unsaturations tended to be more abundant in non-fasted samples. A similar plot of TGs is presented with dots colored by difference in OGTT glucAUC regression slope between non-fasted and fasted samples (Supplementary Fig. 3d). A greater difference in fasted and fed slopes indicates a larger interaction effect between sampling and glucose tolerance.

To filter our data and identify the metabolites that are most associated with glucose tolerance, we integrated both our multivariate model and individual metabolite analyses (Fig. 5e). After filtering, we found 66 metabolites associated to OGTT glucAUC in non-fasted sampling, versus 32 metabolites in fasted sampling. Next, we compared these 66 non-fasted sampled metabolites in Nile rats to a list of metabolites from a meta-analysis of incident type 2 diabetes in humans^36^. We found two polar metabolites and five lipids that are predictive of diabetes in both our Nile rat cohort and in humans: isoleucine, betaine, PC 18:0_20:3 (38:3), SM d39:1, TG 16:0_16:0_16:0 (48:0), TG 16:0_16:0_18:0 (50:0) and TG 56:6 (Supplementary Data Tables 7 and 9). Of the unmatched 59 Nile rat metabolites, 20 were present in the human meta-analysis, but not found to have a significant relative risk for type 2 diabetes, such as glucose (*q* = 0.09), and the remaining 39 metabolites were not listed in the human meta-analysis, including many of the polyunsaturated TGs that featured prominently in Fig. 5b, such as TG 22:6_22:6_22:6. Future studies are needed to determine the relevance of these unmatched metabolites in human diabetes.

### Low replicate variance in non-fasted sampling is reproducible regardless of age and sex

**Fig. 6:**
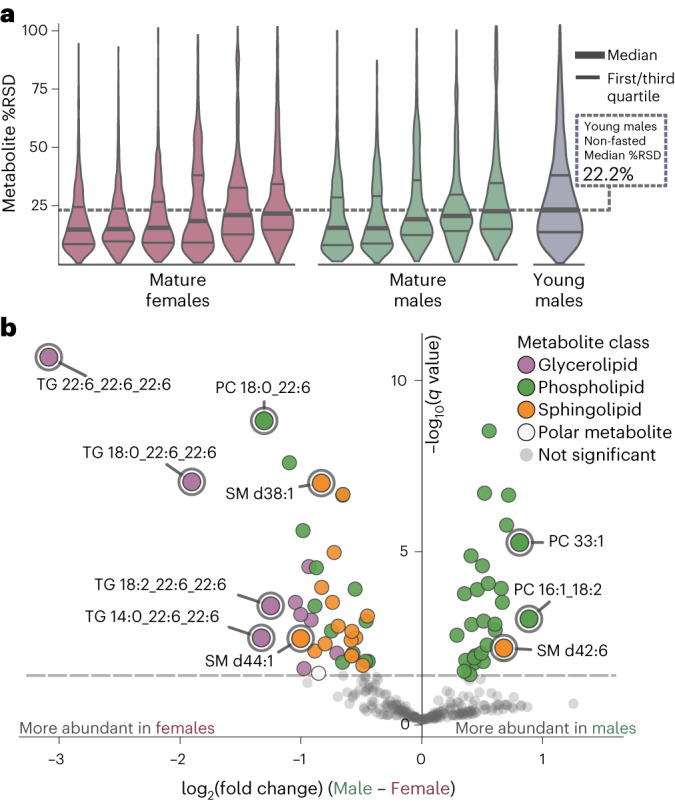
Plasma metabolomics on non-fasted mature male and female Nile rats displaying signs of progressive glucose intolerance shows similar to lower median %RSD compared to young males across triplicate plasma measurements. **a**, A cohort of six female and five male Nile rats underwent triplicate plasma sampling over 3 weeks in the non-fasted state. All 11 Nile rats measured showed lower median plasma metabolite %RSD compared to the median %RSD measurements from all 10 young male Nile rats. **b**, Volcano plot of log_2_ fold change differences between male and female 30-week-old Nile rats for all identified lipids. Polyunsaturated TGs and sphingolipids tend to have higher abundance in females.

Similar to humans, Nile rats can develop diet-induced diabetes throughout a large range of ages. To explore if the low replicate variance in non-fasted sampling is affected by age and sex, we performed a similar analysis to study non-fasted replicate plasma sampling in a mature cohort of male and female Nile rats. To select animals developing diabetes, we collected weekly plasma samples and made weekly RBG measurements from 20 euglycemic Nile rats starting at 24 weeks old and took samples from the first 11 Nile rats (5 males and 6 females) that developed non-fasted hyperglycemia (Supplementary Fig. 4a and Supplementary Data Table 2). Subsequent OGTT of these 11 mature Nile rats revealed OGTT glucAUC values (Supplementary Fig. 4b) similar to the values measured from the previous 10 young male Nile rats. Similar to previously described %RSD measurements (Fig. 3), we assessed %RSD on triplicate non-fasted plasma samples across all lipids and polar metabolites for this mature cohort, and plotted their metabolite %RSD distributions (Fig. 6a and Supplementary Data Tables 4 and 6). In this mature cohort, every Nile rat displayed a lower median metabolite %RSD compared to the 22.2% median %RSD of non-fasted plasma samples in the previous young male cohort. Aggregating %RSDs within the three age and sex groups reveals a statistically significant difference in median %RSD between mature females and mature males (mature males median %RSD 18.4%, mature females median %RSD 16.9%, *P* < 10^−7^). There was also a statistically significant difference in median %RSD in mature males versus young males (*P* < 10^−49^) and mature females versus young males (*P* < 10^−109^). These data support that the low replicate variance in non-fasted plasma sampling is also found in mature Nile rats of both sexes.

Sex differences in type 2 diabetes are well recognized but poorly studied^37,38^. Here we compare plasma metabolite abundances between sexes in our mature cohort. We plot log_2_ fold change of mean abundance between males and females across all annotated metabolites (Fig. 6b). With the exception of SM d42:6, many sphingolipids including SM d44:1 and SM d38:1 were more abundant in females compared to males. This is consistent with human data that shows a similar trend where most SMs are more abundant in females^39,40^. Notably, lipids with the greatest fold change contain polyunsaturated 22:6 fatty acyl, for example, TG 22:6_22:6_22:6 (TG 66:18), TG 18:0_22:6_22:6 (TG 62:12) and PC 18:0_22:6 (PC 40:6) (Supplementary Data Table 8). In summary, the metabolic profiles of the male and female Nile rats during progression of diabetes are highly disparate.

## Discussion

In this study, we used LC–MS to generate the metabolic profile of Nile rats with a spectrum of glucose tolerance spanning euglycemic to overt diabetes and assessed the differences between fasted and non-fasted sampling. In our primary cohort of juvenile male Nile rats, we showed that metabolite measurements in non-fasted samples were more reproducible with lower replicate variance per animal compared to fasted samples. In these rats, non-fasted metabolite measurements were also better than fasted measurements for predicting 12-week glucose tolerance in young male Nile rats. Next we assessed metabolites in the context of glucose tolerance, where we found 66 metabolites highly associated with OGTT glucAUC using a combined approach with multivariable elastic net and individual metabolite linear models. These include isoleucine, betaine, PC 18:0_20:3 (38:3), SM d39:1, TG 16:0_16:0_16:0 (48:0), TG 16:0_16:0_18:0 (50:0) and TG 56:6 that were also found to be significant type 2 diabetes biomarkers in humans. Our findings support non-fasted blood sampling for metabolomics with stronger associations to escalating impaired glucose tolerance and disproved a long-held assumption of higher replicate variance from ad libitum feeding; we anticipate these data will critically inform on future studies in this valuable Nile rat model for diabetes research.

In the past, metabolomics studies in humans have utilized plasma or serum sampled under fasted state^41^. However, a growing number of studies are promoting the use of non-fasted or postprandial sampling for metabolic studies^42,43^. Compared to these studies, our data provides similar conclusions, both in terms of biomarkers^36^ and higher reproducibility of non-fasted versus fasted sampling^20,44^. Metabolite biomarkers found to agree between our work and a human meta-analysis of incident type 2 diabetes biomarkers are metabolites such as isoleucine^45,46^, betaine^47^, TGs in general^48^ and SMs^49^. In addition, we found biomarkers that were not included in the larger human meta-analysis, but have been found in other human diabetes studies, including polyunsaturated lipids SM d40:7, PC 38:7 and PC 40:6 (ref. ^50^). We recognize that fasted sampling is also useful, and contains orthogonal information (vide infra). However, the benefits of non-fasted sampling in animal model studies outweighs fasted sampling, namely the lighter workload in managing animals, lower rates of complications due to fasting and improved reproducibility.

This work is the first plasma metabolomics and lipidomics study in the Nile rat species with several key benefits. First, we performed separate lipidomics and polar metabolomics to provide broader coverage of the diverse molecules present in plasma, from hydrophobic TGs to hydrophilic carbohydrates. Second, we used small amounts of plasma at just 5 µl for each sample. With such small quantities, this opens up avenues for further analysis of plasma in smaller Nile rats, such as in weanlings, enabling the monitoring of plasma metabolites at even younger ages in populations that show early progression of diabetes. In addition, we used two separate machine-learning approaches. The first method uses multivariate regression with regularization to determine a subset of metabolites that work together to predict OGTT glucAUC, while the second approach evaluates each metabolite’s association with OGTT glucAUC. In both approaches we used regression instead of categorical classification; while this approach is uncommon, the methods used here could benefit other diabetes studies that measure continuous variables such as blood glucose, insulin AUC, Homeostatic Model Assessment for Insulin Resistance (HOMA-IR) or hemoglobin A1C (HbA1c). Finally, we performed this study using a study design where each Nile rat underwent replicate sampling under both fasted and non-fasted sampling conditions. This enabled greater statistical power in assessing metabolite replicate variance by using paired statistics between metabolites.

Since our focus was to develop an optimal method for reproducible plasma metabolite measurements in the Nile rat model, this study is limited in its ability to discover diabetes biomarkers due to the small study size and short sampling timeline. In addition, rodent chow is diabetogenic in Nile rats^4,28^ and this choice of diet skews the population toward higher glucose intolerance. Future studies to investigate the underlying mechanisms of glucose tolerance could be enhanced by incorporating food intake and additional metrics of diabetes; however these data were not captured here due to our priority on the metabolomics data. In summary, the method presented in this manuscript enables larger studies that could use metabolomics to explore diabetes progression, analyze the effects of different diets and define the genetic and epigenetic contributions to diabetes in Nile rats.

The Nile rat model is highly valuable for mechanistic studies of type 2 diabetes, with a wide range of phenotypes and propensity to develop diet-induced diabetes on conventional rodent chow. Despite a modest cohort size, the metabolic biomarkers detected here in Nile rats show good agreement with human studies of type 2 diabetes. Importantly, we have strong evidence of low replicate variance in non-fasted sampling supporting the use of non-fasted sampling for future work. Lastly, the LC–MS metabolomics described here enables a broad coverage of metabolites and lipids in a very small volume of plasma. In conclusion, our method is highly suited to reveal complex metabolic changes occurring with progression toward overt diabetes.

## Methods

### Animal studies

All animal experiments were approved by the University of California (protocol number 893), Santa Barbara, Institutional Animal Care and Use Committee, and conducted in accord with the NIH Guide for the Care and Use of Laboratory Animals. The Nile rats were fed ad libitum on a regular rodent diet (Diet 5008; Newco Speciality)^28^, and housed in a 12-h, 10:00 to 22:00, light cycle room. A total of three cohorts of Nile rats were used: ten male Nile rats had blood sampled at 8–10 weeks old (primary dataset), six male and seven female Nile rats had age range between 38 and 42 weeks old (12-h RBG data set) and five males and six females had blood sampled at ages 26–34 weeks old (validation dataset). To perform OGTT, Nile rats were fasted for 16 h from 18:00 to 10:00, and 2 g of dextrose per kilogram body weight was introduced via oral gavage. Fasted plasma samples were collected around 10:00 to 11:00, and non-fasted samples were collected around 15:00 to 16:00, in the middle of the light-on duration. Blood collections were done under fasted and non-fasted conditions in triplicates spaced apart weekly. To minimize the effect of fasting on subsequent non-fasted samples, the Nile rat was allowed to recover for 3 days between the fasted sampling and the next non-fasting sampling. After the last collection, the rats were allowed to recover for 2 weeks before OGTT. All plasma samples were stored at −80 °C. For the OGTT, the OGTT glucAUC and OGTT insAUC for each animal were calculated by trapezoidal integration of the corresponding blood glucose (mg/dL) or blood insulin (ng/dL) at measurement time points of 0, 10, 20, 30, 60 and 120 min. Animals used in this study were not subjected to any previous procedures and have not been genetically modified. Two animals from the validation dataset were excluded because one had an unexpected weight drop and the other developed eye swelling; they were euthanized for humane reasons.

### Glucose and insulin measurements

Blood glucose was measured by a Contour Next glucometer using blood from a tail prick. Plasma insulin was measured using the Ultrasensitive Mouse Insulin ELISA Kit (90080) by Crystal Chem according to standard protocol.

### Lipidomics and metabolomics sample preparation

Plasma samples were thawed once before the second thawing on ice for subsequent lipidomic and metabolomic sample preparation. Each sample of 5 µL of plasma was extracted with 500 µL 6:2:2 *n*-butanol:acetonitrile:water^51,52^. Samples were sonicated in a chilled water bath (QSonica) at an amplitude of 30 for 5 min at 10 °C using time increments of 20 s on/10 s off. Samples were then vortexed for 10 s and then centrifuged at 14,000*g* for 2 min at 4 °C to precipitate the protein. Then 100 µL of extract was dried down in an amber autosampler vial with glass insert by a SpeedVac evaporator (Thermo Scientific). For lipidomics, each extract was resuspended in 50 µL 9:1 methanol:toluene. For metabolomics, each extract was resuspended in 25 µL 1:1 acetonitrile:water then analyzed on the mass spectrometer. For both LC–MS methods, run order of plasma samples was randomized to minimize confounding effects of instrument variance over time.

### HILIC–LC–MS metabolomics

Sample analysis was performed on a ZIC-pHILIC HPLC column held at 50 °C (100 mm × 2.1 mm × 1.7 μm particle size; Millipore) using a Vanquish Binary Pump (150 μL/min flow rate; Thermo Scientific). Mobile phase A consisted of 10 mM ammonium acetate in acetonitrile:H_2_O (10:90, v/v) containing 0.1% ammonium hydroxide. Mobile phase B consisted of 10 mM ammonium acetate in acetonitrile:H_2_O (95:5, v/v) containing 0.1% ammonium hydroxide. Mobile phase B was initially held at 95% for 2 min and then decreased to 30% over 18 min. Mobile phase B was held for 6 min at 35%, then raised to 95% over 1 min. The column was re-equilibrated at 95% mobile phase B for 8 min. Two microliters of extract was injected by a Vanquish Split Sampler HT autosampler (Thermo Scientific).

The LC system was coupled to a Q Exactive-HF Orbitrap mass spectrometer through a heated electrospray ionization (HESI II) source (Thermo Scientific). Source conditions were as follows: HESI II and capillary temperature at 350 °C, sheath gas flow rate at 40 units, aux gas flow rate at 15 units, sweep gas flow rate at 1 unit, spray voltage at |3.0 kV| for both positive and negative modes, and S-lens RF at 50.0 units. The MS was operated in a polarity switching mode acquiring positive and negative full MS and MS2 spectra (Top10) within the same injection. Acquisition parameters for full MS scans in both modes were 60,000 resolution, 1 × 10^6^ automatic gain control (AGC) target, 100 ms ion accumulation time (max IT), and 70–900 *m*/*z* scan range. MS2 scans in both modes were then performed at 45,000 resolution, 1 × 10^5^ AGC target, 100 ms max IT, 1.0 *m*/*z* isolation window, stepped normalized collision energy at 20, 30, 40 and a 30.0 s dynamic exclusion.

### Reversed phase LC–MS lipidomics

Ten microliters of sample extract was injected via Vanquish Split Sampler HT autosampler (Thermo Scientific) onto an ACQUITY CSH C18 column held at 50 °C (100 mm × 2.1 mm × 1.7 μm particle size; Waters) using a Vanquish Binary Pump (400 μL/min flow rate; Thermo Scientific). A reversed phase gradient length of 30 min was used to separate the lipids, using mobile phase A, consisting of 10 mM ammonium acetate in acetonitrile:water (70:30, v/v) containing 250 μL/L acetic acid, and mobile phase B, consisting of 10 mM ammonium acetate in isopropanol:acetonitrile (90:10, v/v) with the same additives. Mobile phase B was initially held at 2% for 2 min and then increased to 30% over 3 min. Mobile phase B was further increased to 50% over 1 min, then raised to 85% over 14 min, and finally raised to 99% over 1 min and held at 99% for 7 min. Mobile phase B was then decreased to 2% over 0.25 min, and the column was re-equilibrated with mobile phase B at 2% for 1.75 min before the next injection.

The LC system was coupled online to a Q Exactive-HF Orbitrap mass spectrometer through a heated electrospray ionization (HESI II) source (Thermo Scientific). In both ionization modes, the HESI II and capillary temperature, spray voltage, S-lens RF level, sheath gas, aux gas and sweep gas were held at 300 °C, |3.5 kV | , 90.0 units, 25 units, 15 units and 5 units, respectively. The MS was operated in a polarity switching mode acquiring positive and negative full MS and MS2 spectra (Top2) within the same injection. Acquisition parameters for full MS scans in both modes were 17,500 resolution, 1 × 10^6^ AGC target, 100 ms ion accumulation time (max IT), and 200–1,600 *m*/*z* scan range. Data-dependent MS2 scans in both modes were then performed at 17,500 resolution, 1 × 10^5^ AGC target, 50 ms max IT, 1.0 *m*/*z* isolation window, stepped normalized collision energy at 20, 30, 40 and a 10.0 s dynamic exclusion.

### LC–MS data processing

Reversed phase LC–MS raw lipidomics data were processed in Compound Discoverer 3.1 (Thermo Scientific) in conjunction with LipiDex^53^. In brief, MS1 scans from 100 Da to 5,000 Da precursor mass as well as retention time of 0.4 min to 21 min were extracted and aligned, using alignment parameters as follows: 0.2 min retention time tolerance, 10-ppm mass, a minimum peak intensity of 5 × 10^5^, a maximum peak width of 0.25 min, and a minimum signal-to-noise ratio of 1.5, to form distinct chromatographic profiles, or compound groups. From the chromatographic features that were at least three-fold greater in intensity than blanks, the consequent MS2 features were searched against an in silico generated lipid spectral library. Compounds were annotated only if the corresponding MS2 fulfilled the following requirements: a minimum lipid spectral purity of 75% from co-eluting isobaric lipids that elute within a 3.5 median absolute retention time deviation from each other, a minimum MS2 spectral match dot product of 500, a minimum MS2 spectral match reverse dot product of 700, and found within at least two processed files. For individual fatty acid substituents that could not be resolved, the identifications were generated with the sum of the fatty acid substituents. Features were removed from further consideration if the %RSD values from quality control replicates were greater than 30%.

Hydrophilic interaction liquid chromatography (HILIC)–LC–MS raw metabolomics data were processed using the default workflow Untargeted Metabolomics using Online Databases, mzLogic and Molecular Networks in Compound Discoverer 3.3 (Thermo Scientific). Annotations for polar metabolites were derived from MS2 libraries using authentic standards, or from mzCloud library matching followed by manual validation of identifications using combined evidence from MS2 library matching score greater than 80, in addition to retention time and the presence of metabolite in databases of plasma metabolites^54^. Polar metabolite features were removed if %RSD of replicate quality control was >30%.

### Statistical analysis

Data processing was performed in Python 3.7 with the following packages and versions: statsmodels 0.13.2; shap 0.41.0; scikit-learn 1.0.2; scipy 1.7.3; pandas 1.3.5; numpy 1.21.6; networkx 2.6.3; matplotlib 3.5.2; matplotlib-venn 0.11.5; seaborn 0.11.2.

PCA was calculated on all 60 plasma samples (10-week-old males), combining both annotated and unannotated LC–MS chromatographic features from reversed phase lipidomics method and HILIC polar metabolomics method. Points represent samples and were plotted based on principal components 1 and 2. Samples were labeled according to the Nile rat label and whether the sample was fasted or non-fasted. In the process of PCA, the fasted 9-week sample from Nile rat A was found to lie within the non-fasted cluster. Further analysis of the metabolite profile revealed elevated AAs with an outlier effect of >2 standard deviations compared to other fasted samples, leading us to remove this sample from PCA visualization and discard this sample from further downstream analyses. The heat map in Fig. 2d was generated using Python seaborn. Each column is one Nile rat’s annotated metabolite profile in either fasted or non-fasted conditions, averaged across triplicate sampling weeks. Rows are one annotated metabolite, and log_2_ fold change is given as the difference between the triplicate averaged log_2_ abundance and the mean log_2_ abundance of all fasted plasma samples. Rows were hierarchically clustered using method complete linkage with Euclidean (L2 norm) distance metric.

%RSD was calculated for each metabolite feature, within each sampling method (non-fasted and fasted), within each Nile rat, in both Nile rat cohorts (young males and mature males/females). The calculation uses metabolite log_2_ abundances to find the standard deviation of triplicate sampling across 3 weeks divided by the mean of these three values. Young male Nile rat A was excluded from %RSD calculations due to discarding outlier week of fasted sampling. Significance testing between young male non-fasted and fasted metabolite %RSDs was performed using Wilcoxon signed rank test on paired %RSD values among the metabolite groups using scipy wilcoxon function. Calculated *P* values from Wilcoxon signed rank were corrected for false discovery rate by Bonferroni method using statsmodels multipletests function.

Multivariate machine learning models were trained using the associated sklearn method (LinearRegression, Lasso, Ridge, ElasticNet, PLSRegression and RandomForestRegressor). Cross-validation was performed using sklearn cross_validate with n_repeats of 200 and n_splits of 6, with random seed set identically for all six models ensuring the same training data. Competing models for non-fasted and fasted sampling were trained on all young male non-fasted and fasted plasma samples, and the median *R*^2^ value from all 1,200 folds were presented. At each fold, the β coefficient of each metabolite feature was recorded. Metabolite importance is calculated as the average β coefficient across all 1,200 folds divided by the maximum average β coefficient of all metabolites. Normalized importances were then calculated by taking the absolute value of the importance to set each metabolite’s normalized importance value between 0 and 1. Individual metabolite linear regressions and *R*^2^ values of OGTT glucAUC versus log_2_ abundance (used in Fig. 4d) were calculated using all non-fasted and fasted plasma samples, with the dots on the plots representing the mean log_2_ abundance from each Nile rat.

Throughout the text, the term individual metabolite linear models is used, which is defined as the regression model given by equation (1).

1 $$\begin{array}{l}{{\mathrm{Metabolite}}}\,\log_2\,{{\mathrm{abundance}}} \sim {{\mathrm{OGTT}}}\,{{\mathrm{glucAUC}}}\\+\,{{\mathrm{sampling}}}\,+\,{{\mathrm{sampling}}}:{{\mathrm{OGTT}}}\,{{\mathrm{glucAUC}}}\end{array}$$

Significance testing was performed, each of the three terms in equation (1) using likelihood ratio test in statsmodels ols function. *P* values for each of the three terms were corrected across all metabolites using Benjamini–Hochberg false discovery rate correction using statsmodels fdrcorrection. Resulting *q* values were significant at a value of less than 0.05. Each metabolite also underwent linear regression of OGTT glucAUC versus log_2_ abundance within each sampling method, and resulting *P* values for the effect size (regression slope value; used in Fig. 5b) were also corrected for multiple testing using the Benjamini–Hochberg method, with *q* values significant at less than 0.05.

### Reporting summary

Further information on research design is available in the Nature Portfolio Reporting Summary linked to this article.

## Online content

Any methods, additional references, Nature Portfolio reporting summaries, source data, extended data, supplementary information, acknowledgements, peer review information; details of author contributions and competing interests; and statements of data and code availability are available at 10.1038/s41684-023-01268-0.

### Supplementary information

Supplementary Information Supplementary Figs. 1–4.

Reporting Summary

Supplementary Data Supplementary Data Tables 1–9.

## Data Availability

All MS files are available in the public repository MassIVE under accession number MSV000091033.

## Code availability

Code for analysis and figures is contained in GitHub repository at https://github.com/benton-anderson/nile_rat_multiomics
