# Supplementary material for: Plasma metabolomics supports non-fasted sampling for metabolic profiling across a spectrum of glucose tolerance in the Nile rat model for type 2 diabetes
Source: Lab Anim (NY). 2023 Oct 19;52(11):269–77. doi: 10.1038/s41684-023-01268-0 (PMC10611569; doi:10.1038/s41684-023-01268-0)
Supplement: Supplementary file 1 — Supplementary Figs. 1–4. [file 41684_2023_1268_MOESM1_ESM.pdf]

nature portfolio

https://doi.org/10.1038/s41684-023-01268-0

## Supplementary information

# Plasma metabolomics supports non-fasted sampling for metabolic profiling across a spectrum of glucose tolerance in the Nile rat model for type 2 diabetes

In the format provided by the authors and unedited

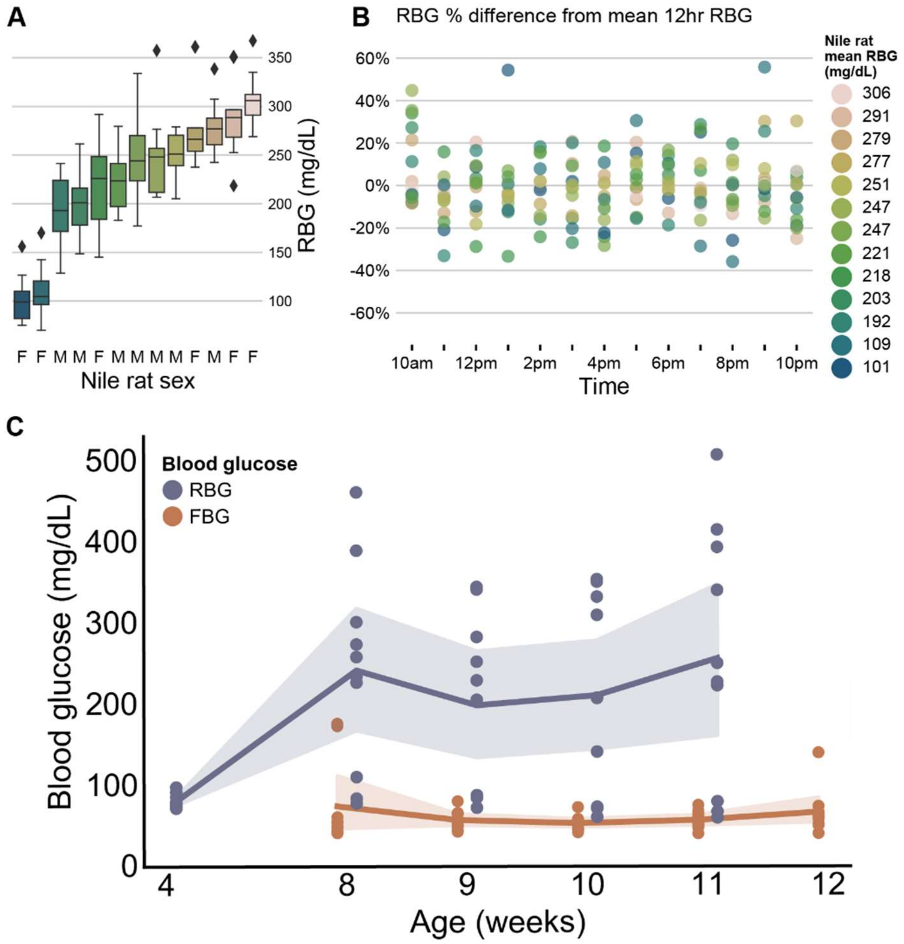


**Supplementary Fig. 1 - Nile rat hourly RBG testing across active hours shows no significant difference in variance across mean RBGs, and no significant difference in average RBG throughout the day**

(**A**) Boxplots show distribution of random blood glucose (RBG) measurements taken every 1 hour over 12 hours from 10 am to 10 pm in a cohort of 6 female and 7 male Nile rats (age 38-42 weeks). Significance testing using Levene's test reveals no significant difference in the variance among all Nile rats' RBG 12-hour distributions (*p=0.99*). (**B**) Scatterplot of RBG measurements from all 13 Nile rats, grouped by hour of day. Dots are colored by Nile rat's mean RBG value from the 12 hour period. RBG Percent difference from mean 12 hour RBG is calculated within each animal for all hourly measurements as [(hourRBG - meanRBG) / meanRBG] * 100%. Significance testing using one-way ANOVA shows no significant difference among hourly RBGs (*p=0.14*). The appearance of increased RBGs at 10 am could be attributable to the dawn phenomenon[1] of heightened morning RBG values, which has been observed in humans. (**C**) Young male Nile rats in the main study cohort underwent random and fasted blood glucose (RBG and FBG) measurements at different ages. Shaded areas indicate 95% bootstrapped confidence intervals for each week's blood glucose measurements.

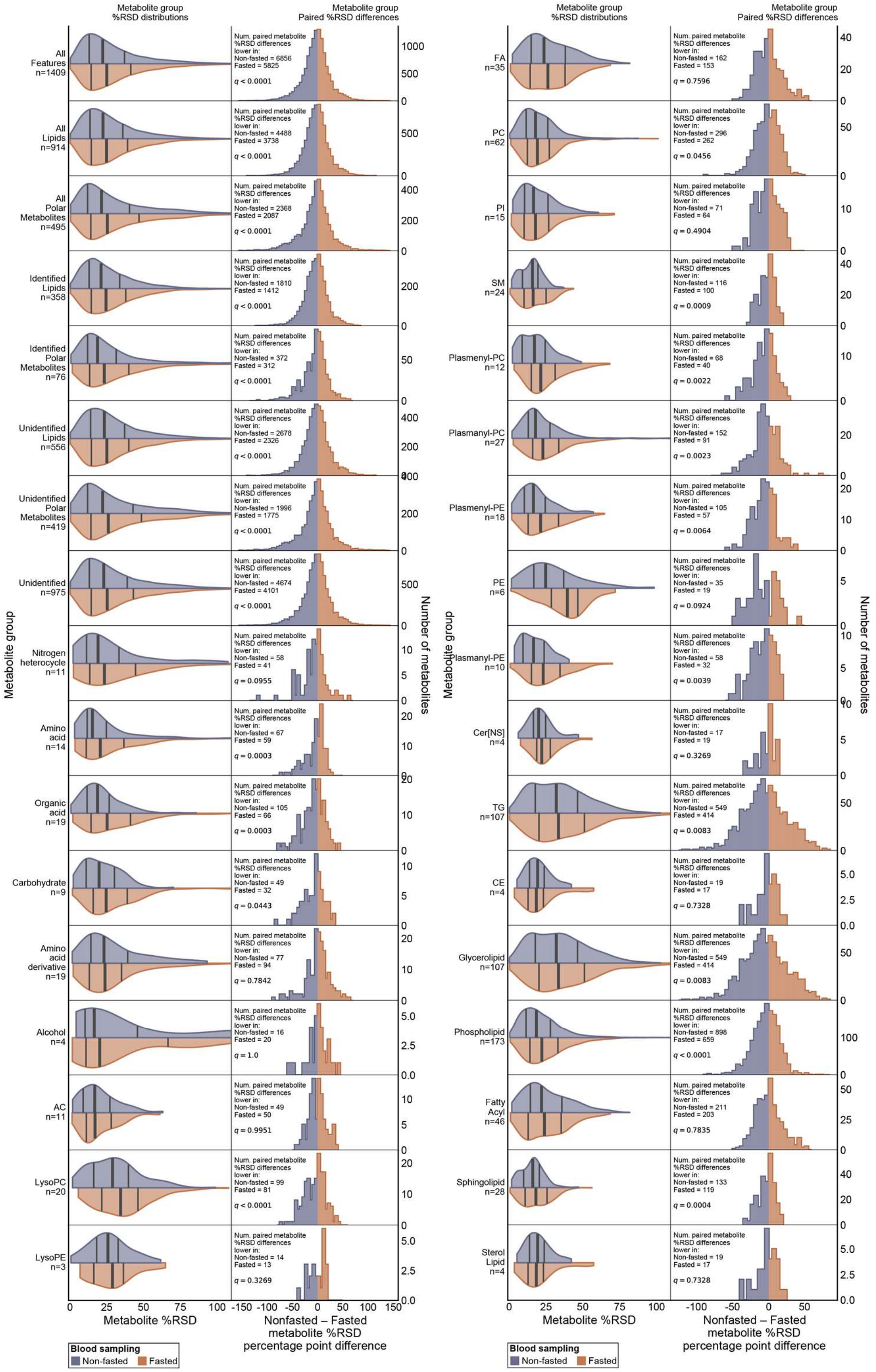


**Supplementary Fig. 2** - Distributions for %RSDs among levels of metabolite groupings are presented. Split violin plots give the distributions of non-fasted and fasted Nile rat plasma samples. Histograms show the percentage point difference between %RSD for each metabolite between the two methods. Significance testing on paired %RSD measurements was performed using Wilcoxon signed rank test. *p*-values were corrected using Benjamini-Hochberg false discovery correction. *q*-values below 0.05 were considered significant.

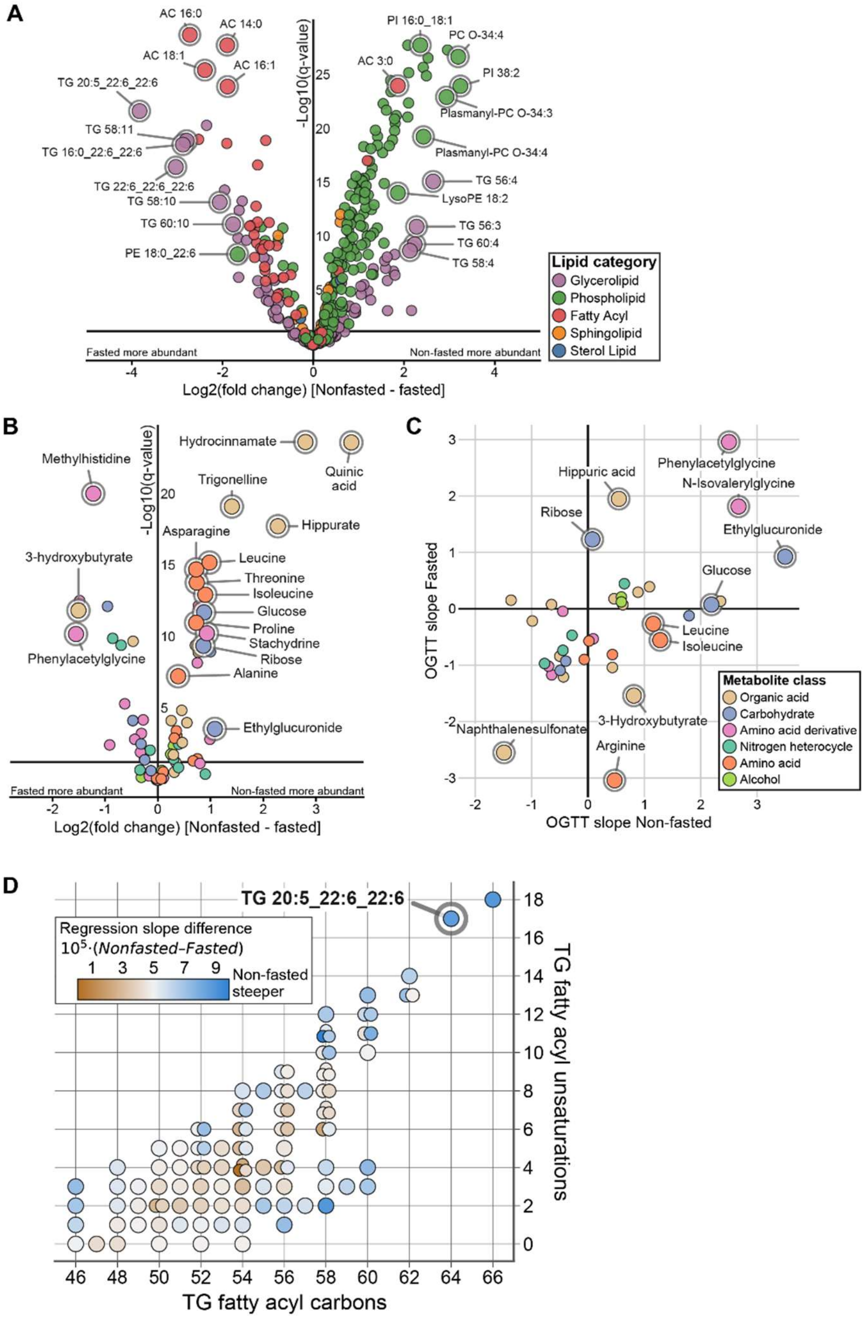


**Supplementary Fig. 3 - Lipids and polar metabolites volcano plots. Fasted vs. non-fasted regression slope plot for polar metabolites.** (**A**) Volcano plot showing log2 fold change of non-fasted samples minus fasted samples for all annotated lipids, with select lipids highlighted. (**B**) Volcano plot showing log2 fold change of non-fasted samples minus fasted samples for all annotated polar metabolites, with select polar metabolites highlighted. (**C**) Plot of regression slopes in fasted versus non-fasted individual metabolite linear models. Metabolites with regression slope effect size *q*-value > 0.1 have been excluded. (**D**) Similar to Figure 5D, all annotated TGs are separated by fatty acyl carbon counts and unsaturations, with dots colored by the difference in OGTT glucAUC regression slope between non-fasted samples and fasted samples.

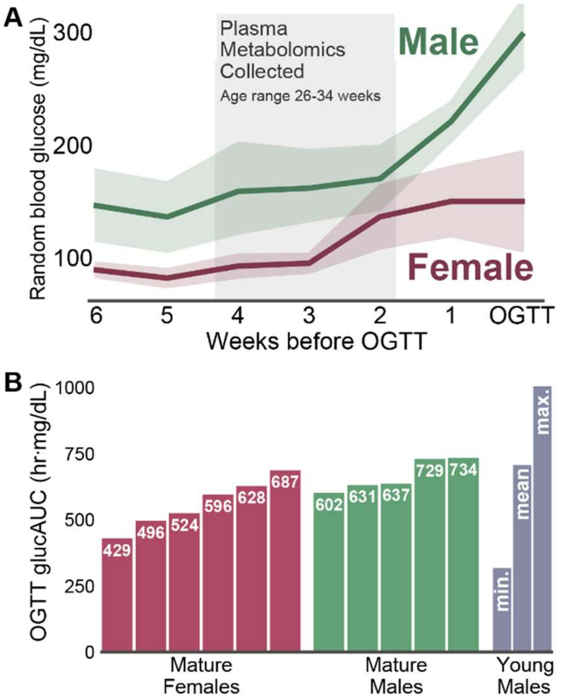


**Supplementary Fig. 4 -** (**A**) A cohort of mature Nile rats were monitored weekly for signs of elevated random blood glucose, with concurrent non-fasted blood sampling. Triplicate plasma samples for %RSD measurement were selected in a 3 week range prior to elevated RBG. The average age at sampling was 30 weeks, with minimum sampling age 26 weeks and maximum sampling age 34. (**B**) Subsequent OGTT glucAUC testing on the cohort of mature Nile rats revealed a range of values that fall within the range of young males assessed earlier in the study, indicating a range of impaired glucose tolerances.

1. Thirty Years of Research on the Dawn Phenomenon: Lessons to Optimize Blood Glucose Control in Diabetes | Diabetes Care | American Diabetes Association. https://diabetesjournals.org/care/article/36/12/3860/33148/Thirty-Years-of-Research-on-the-Dawn-Phenomenon.
