# Supplementary material for: Plasma metabolomics supports non-fasted sampling for metabolic profiling across a spectrum of glucose tolerance in the Nile rat model for type 2 diabetes
Source: Lab Anim (NY). 2023 Oct 19;52(11):269–77. doi: 10.1038/s41684-023-01268-0 (PMC10611569; doi:10.1038/s41684-023-01268-0)
Supplement: Supplementary file 2 — Reporting Summary [file 41684_2023_1268_MOESM2_ESM.pdf]

nature research

Corresponding author(s): Huishi Toh
Katherine Overmyer

Last updated by author(s): Sep 7, 2023

Reporting Summary

Nature Research wishes to improve the reproducibility of the work that we publish. This form provides structure for consistency and transparency in reporting. For further information on Nature Research policies, see Authors & Referees and the Editorial Policy Checklist.

Statistics

For all statistical analyses, confirm that the following items are present in the figure legend, table legend, main text, or Methods section.

n/a	Confirmed	
☐	☒	The exact sample size (n) for each experimental group/condition, given as a discrete number and unit of measurement
☐	☒	A statement on whether measurements were taken from distinct samples or whether the same sample was measured repeatedly
☐	☒	The statistical test(s) used AND whether they are one- or two-sided<br>*Only common tests should be described solely by name; describe more complex techniques in the Methods section.*
☐	☒	A description of all covariates tested
☐	☒	A description of any assumptions or corrections, such as tests of normality and adjustment for multiple comparisons
☐	☒	A full description of the statistical parameters including central tendency (e.g. means) or other basic estimates (e.g. regression coefficient) AND variation (e.g. standard deviation) or associated estimates of uncertainty (e.g. confidence intervals)
☐	☒	For null hypothesis testing, the test statistic (e.g. F, t, r) with confidence intervals, effect sizes, degrees of freedom and P value noted<br>*Give P values as exact values whenever suitable.*
☒	☐	For Bayesian analysis, information on the choice of priors and Markov chain Monte Carlo settings
☒	☐	For hierarchical and complex designs, identification of the appropriate level for tests and full reporting of outcomes
☒	☐	Estimates of effect sizes (e.g. Cohen's d, Pearson's r), indicating how they were calculated

Our web collection on statistics for biologists contains articles on many of the points above.

Software and code

Policy information about availability of computer code

Data collection	Thermo Fisher Xcalibur with LC-MS Method editor, 3.1
Data analysis	Data processing was performed in Python 3.7 with the following packages and versions: statsmodels 0.13.2; shap 0.41.0; scikit-learn 1.0.2; scipy 1.7.3; pandas 1.3.5; numpy 1.21.6; networkx 2.6.3; matplotlib 3.5.2; matplotlib-venn 0.11.5; seaborn 0.11.2. Processing code located at https ://gith ub .com/benton-anderson/n ile_rat_mu ltiomics

For manuscripts utilizing custom algorithms or software that are central to the research but not yet described in published literature, software must be made available to editors/reviewers. We strongly encourage code deposition in a community repository (e.g. GitHub). See the Nature Research guidelines for submitting code & software for further information.

Data

Policy information about availability of data

All manuscripts must include a data availability statement. This statement should provide the following information, where applicable:

- Accession codes, unique identifiers, or web links for publicly available datasets
- A list of figures that have associated raw data
- A description of any restrictions on data availability

All mass spectrometry files are available in the public repository MassIVE under accession number MSV000091033. Code for analysis and figures is contained in GitHub repository at https://github.com/benton-anderson/nile_rat_multiomics

Field-specific reporting

Please select the one below that is the best fit for your research. If you are not sure, read the appropriate sections before making your selection.

☒ Life sciences ☐ Behavioural & social sciences ☐ Ecological, evolutionary & environmental sciences

For a reference copy of the document with all sections, see nature.com/documents/nr-reporting-summary-flat.pdf

Life sciences study design

All studies must disclose on these points even when the disclosure is negative.

Sample size	Sample sizes detailed in Methods. Samples of 10, 13 and 11 Nile rats were chosen due to availability of suitable animals within the colony, and also due to mass spectrometry instrument availability.
Data exclusions	8-10 week male Nile rat (code 1091} week 9 fasted plasma sample was excluded due to outlier quantitations of metabolites. On PCA analysis the sample appeared to cluster with the non-fasted samples, and in per-metabolite analysis the sample's quantitations were consistently an outlier.
Replication	See Figure 6 content and Discussion.
Randomization	Samples were randomized in order during LC-MS runs.
Blinding	Blinding not necessary in animal handling due to all animals undergoing identical treatments of fasting and non-fasting. Data labels were not blinded during data analysis.

Reporting for specific materials, systems and methods

We require information from authors about some types of materials, experimental systems and methods used in many studies. Here, indicate whether each material, system or method listed is relevant to your study. If you are not sure if a list item applies to your research, read the appropriate section before selecting a response.

Materials & experimental systems

n/a	Involved in the study	
☒	☐	Antibodies
☒	☐	Eukaryotic cell lines
☒	☐	Palaeontology
☐	☒	Animals and other organisms
☒	☐	Human research participants
☒	☐	Clinical data

Methods

n/a	Involved in the study	
☒	☐	ChIP-seq
☒	☐	Flow cytometry
☒	☐	MRI-based neuroimaging

Animals and other organisms

Policy information about studies involving animals; ARRIVE guidelines recommended for reporting animal research

Laboratory animals	Nile rats (Arvicanthis niloticus), males and females, aged 4 weeks to 1 year old.
Wild animals	Study did not involve wild animals.
Field-collected samples	Study did not involve samples collected from the field.
Ethics oversight	All animal experiments were approved by the University of California (Protocol Number 893), Santa Barbara, Institutional Animal Care and Use Committee, and conducted in accord with the NIH Guide for the Care and Use of Laboratory Animals.

Note that full information on the approval of the study protocol must also be provided in the manuscript.
